# A Low Memory Requirement MobileNets Accelerator Based on FPGA for Auxiliary Medical Tasks

**DOI:** 10.3390/bioengineering10010028

**Published:** 2022-12-24

**Authors:** Yanru Lin, Yanjun Zhang, Xu Yang

**Affiliations:** 1 School of Integrated Circuits and Electronics, Beijing Institute of Technology, No. 5, South Street, Zhongguancun, Haidian District, Beijing 100081, China; 2 School of Cyberspace Science and Technology, Beijing Institute of Technology, No. 5, South Street, Zhongguancun, Haidian District, Beijing 100081, China; 3 School of Computer Science and Technology, Beijing Institute of Technology, No. 5, South Street, Zhongguancun, Haidian District, Beijing 100081, China

**Keywords:** convolutional neural network, FPGA, hardware accelerator, MobileNetV2, auxiliary medical tasks

## Abstract

Convolutional neural networks (CNNs) have been widely applied in the fields of medical tasks because they can achieve high accuracy in many fields using a large number of parameters and operations. However, many applications designed for auxiliary checks or help need to be deployed into portable devices, where the huge number of operations and parameters of a standard CNN can become an obstruction. MobileNet adopts a depthwise separable convolution to replace the standard convolution, which can greatly reduce the number of operations and parameters while maintaining a relatively high accuracy. Such highly structured models are very suitable for FPGA implementation in order to further reduce resource requirements and improve efficiency. Many other implementations focus on performance more than on resource requirements because MobileNets has already reduced both parameters and operations and obtained significant results. However, because many small devices only have limited resources they cannot run MobileNet-like efficient networks in a normal way, and there are still many auxiliary medical applications that require a high-performance network running in real-time to meet the requirements. Hence, we need to figure out a specific accelerator structure to further reduce the memory and other resource requirements while running MobileNet-like efficient networks. In this paper, a MobileNet accelerator is proposed to minimize the on-chip memory capacity and the amount of data that is transferred between on-chip and off-chip memory. We propose two configurable computing modules: Pointwise Convolution Accelerator and Depthwise Convolution Accelerator, to parallelize the network and reduce the memory requirement with a specific dataflow model. At the same time, a new cache usage method is also proposed to further reduce the use of the on-chip memory. We implemented the accelerator on Xilinx XC7Z020, deployed MobileNetV2 on it, and achieved 70.94 FPS with 524.25 KB on-chip memory usage under 150 MHz.

## 1. Introduction

Currently, convolutional neural networks (CNNs) have become an important part of many applications due to their superior performance, especially in the parts related to computer vision, such as object detection [1] and image classification [2]. In the medical domain, CNNs have also been used to achieve many tasks such as MRI image classification [3].

Medical tasks have their own specific characteristics, some of them require only a very high accuracy and can ignore resource requirements. However, many other auxiliary tasks under human supervision are supposed to be deployed into portable devices such as small cameras or monitors connected with a scope to classify histopathological changes and run in real-time. Many other medical checks or cares also need to run on small devices and in real-time, such as mask checks and patient care. However, for standard CNNs [4,5,6], the high-power consumption and the huge number of parameters and computation make it difficult for them be deployed in small devices. Thus, various efficient CNNs with remarkable results have been proposed, such as MobileNetV2 [7], ShuffleNetV2 [8], and Xception [9]. They reduce the memory requirement and the amount of computation notably while keeping a relatively high accuracy.

Thanks to the achievement of efficient CNNs, many relevant medical works have been proposed by using efficient CNNs and have achieved good results. For human-supervised situations, such applications could help to identify pathologic tissues quickly and provide hints for doctors or point out neglected parts. It allows for quicker and more accurate diagnosis. For example, a real-time analysis of colonoscopies by adapting MobileNet is proposed by [10]. This network could be deployed into a small computer connected to the colonoscope and show results on the monitor in order to provide real-time information to manipulators. This kind of computer-aided diagnosis also allows patients to obtain reference reports quickly. Skin lesions may cause skin cancer and other harmful effects. However, it could be a treatable disease and increase the survival rate if the skin lesion is detected in an early stage. Thus, the authors in [11] use MobileNet to perform skin lesion classification; this network could be deployed into specific portable devices or even smartphones to allow patients to conduct a computer-aided diagnosis early and ask for suggestions from doctors early. For human-unsupervised situations, many methods have been designed to provide medical help. Hand gestures are an important way of allowing disabled patients to interact with things. Such a system is proposed by [12]; they built a hand gesture recognition system using MobileNetV2 for disabled patients as a notification system. Furthermore, to help fight against COVID-19, a face mask detection method using MobileNet [13] was proposed to remind people to wear their masks when coming into indoor places. These applications could be implemented into more small devices such as surveillance cameras.

Though efficient CNNs such as MobileNets could reduce resource requirements. We still need an accelerator to achieve high resource utilization since general processors cannot fit CNNs’ structures perfectly. FPGA or ASIC could be a choice to run and accelerate CNNs. FPGA has the ability to support frequent updating of the architecture of CNN models; therefore, we will focus on FPGA-based efficient CNN accelerators.

Most edge devices and small FPGAs only have a few kilobytes or less of on-chip memory. Adding a high-capacity off-chip memory such as DRAM and storing all data in it could be a good choice because DRAM can store gigabytes of data. However, off-chip memory has higher latency compared with one or two cycles latency of on-chip memory. Off-chip memory also consumes two orders of magnitude higher energy than small on-chip memory [14]. Hence, we propose a high-performance accelerator based on FPGA for MobileNets, which aims to minimize the usage of on-chip memory and data transfer between on-chip memory and off-chip memory. The accelerator is also designed to minimize the requirement of other resources in order to be deployed on small devices.

The main contributions of this work are:

1. A high-performance CNN hardware accelerator is proposed where operations are processed in parallel in the Pointwise Convolution Accelerator and Depthwise Convolution Accelerator individually. The Depthwise Convolution Accelerator could be pipelined after the Pointwise Convolution Accelerator or bypassed to match with depthwise separable convolutions in MobileNets, leading to less off-chip memory access and higher inference speed.
2. A changeable number of channels in the Pointwise Convolution Accelerator could reduce both memory and computation resource requirement of blocks behind the Pointwise Convolution Accelerator and maximize utilization of existing resources.
3. The usage of on-chip memory and data interaction between on-chip memory and off-chip memory can be minimized by using a switchable ping-pong buffer structure and reorganized dataflow to further reduce memory requirement.

## 2. Related Works

Firstly, an overview is provided by [14], showing the basic components of NN, its history, and why NNs are important. It also discusses various hardware platforms and architectures that are proposed to support NNs and how they help to reduce computation costs solely via hardware design changes or joint hardware design and NN algorithm changes.

Traditional CNNs usually require large storage and computation resources, which makes it difficult to deploy them into FPGAs. Though many architectures are proposed to conquer parts of these difficulties such as [15,16], they store parameters into the on-chip memory to deal with memory bandwidth limitation in order to accelerate CNNs. Nevertheless, it is still hard to obtain high performance. So many recent accelerators choose depthwise separable convolution based networks as their target network to reduce storage requirements. The method proposed in [17] implements a variant network of ShuffleNetV2 on the ZU3EG platform and achieves 96.5 FPS and 159 BRAM usage. It can achieve 68.47% top-1 accuracy on the ImageNet classification task. Another work [18], uses methods such as pruning to reduce redundant parameters and operations of MobileNet and deploy it on the ZU9EG platform. Results show that it achieves 64.6% top-1 accuracy on the ImageNet classification task with 25× fewer parameters compared with AlexNet.

For MobileNetV2 specifically, many hardware accelerators have also been proposed. The authors of [19] propose a single computing engine that can deal with both pointwise convolution and depthwise convolution efficiently. However, under some conditions, the computing process of depthwise convolution cannot start until the previous pointwise convolution has been completed. It achieves 266.6 FPS on Arria10, and the feature map cache is 24.5 Mb. An accelerator aiming to boost inverted residual layers is proposed by [20]. It introduces a two-layer (pointwise–depthwise) pipeline to maximize resource utilization. Furthermore, it also analyzes the workload of pointwise convolution and depthwise convolution to determine the best configuration of its accelerator. It achieves 205.3 FPS on Xilinx UltraScale ZU2 under 430 MHz. The total memory usage is 145 BRAM. An ultra-high throughput design is achieved by [21]. Many more DSPs are used to reduce computing latency. It also uses a two-layer pipeline to increase the throughput. Two of the same accelerators are implemented on the FPGA with independent memory that is interconnected to further improve performance. It achieves 1050 FPS on Arria10 under 200 MHz. The cache usage is 15.3 Mb. A hybrid dataflow structure is proposed in [22], which focuses on improving resource utilization and reducing the memory requirement by using suitable dataflow schemes for each different layer.

All these accelerators focus on basic computing components of MobileNets and achieve significant results. However, they either neglect to optimize the memory model to fit MobileNets’ structures or design excessively complex structures to optimize each layer, which ignores the characteristics of MobileNets. In this paper, the proposed method will focus on both computing and memory architectures in order to minimize memory and other resource requirements and maintain a relatively high efficiency.

## 3. Background

In this Section, the depthwise separable convolution (DSC) and MobileNets will be introduced. In Section 3.1, the mechanism of DSC will be introduced in detail, and the benefits of DSC compared with the standard convolution will also be calculated and given as equations. In Section 3.2, we introduce MobileNet and MobileNetV2 as examples of efficient networks that are based on DSC. Their specific structures and the final performances will be also given.

### 3.1. Depthwise Separable Convolution

**Figure 1:**
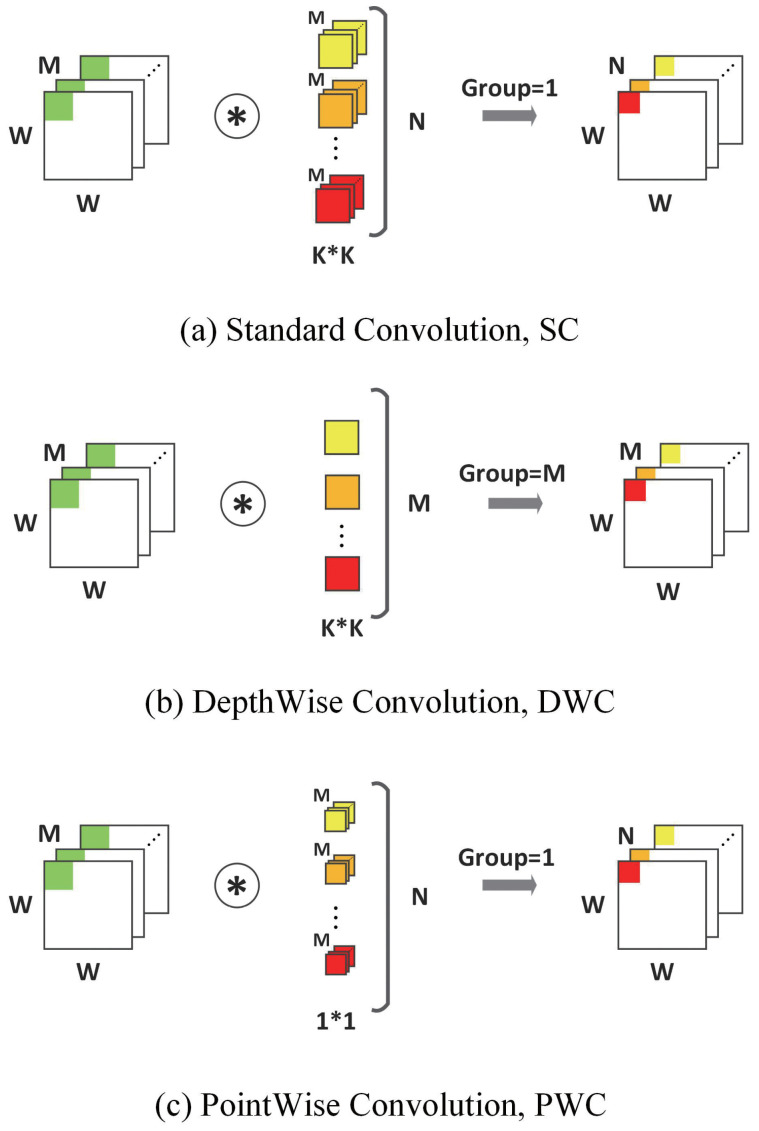
Standard convolution and depth separable convolution.

Depthwise separable convolution was first introduced in [9], it replaces the standard convolution (SC) with the depthwise separable convolution (DSC). DSC is composed of a depthwise convolution (DWC) and a pointwise convolution (PWC). Specifically, DWC performs a convolution with the same kernel size and stride of SC but for each input channel individually. This means that the sizes of input channels, output channels, and kernel numbers are always the same. Furthermore, PWC performs a standard convolution with a 1×1 kernel size. Figure 1a demonstrates how an SC works, and Figure 1b,c demonstrates how a DWC works. This kind of replacement is proved by [23] and can be mathematically approximately equal to SC.

One notable benefit of using DSC is the decrease in the number of operations and parameters. As it is shown in Figure 1, assuming that we have the input feature map with size W∗W∗M and kernel size K∗K∗M∗N, where M is the size of input channels and N is the size of output channels, for an SC whose stride is 1; the size of output feature map is W∗W∗N; and the number of operations $O_{SC}$ and parameters $W_{SC}$ is:

(1) $$O_{SC}=W*W*K*K*M*N$$

(2) $$W_{SC}=K*K*M*N$$

While in the case of DSC, the DWC’s total number of operations and parameters are referred as $O_{DWC}$ and $W_{DWC}$; $O_{PWC}$ and $W_{PWC}$ refer to the same things for PWC. Hence the total number of operations $O_{DSC}$ and parameters $W_{DSC}$ is:

(3) $$O_{DSC}=O_{DWC}+O_{PWC}=W*W*K*K*M+W*W*M*N$$

(4) $$W_{DSC}=W_{DWC}+W_{PWC}=K*K*M+M*N$$

As a result, we can see the reduction factor of parameters $F_{W}$ and operations $F_{O}$ is:

(5) $$F_{W}=\frac{W_{DSC}}{W_{SC}}=F_{O}=\frac{O_{DSC}}{O_{SC}}=\frac{1}{N}+\frac{1}{K^{2}}$$

### 3.2. MobileNet and MobileNetV2

**Figure 2:**
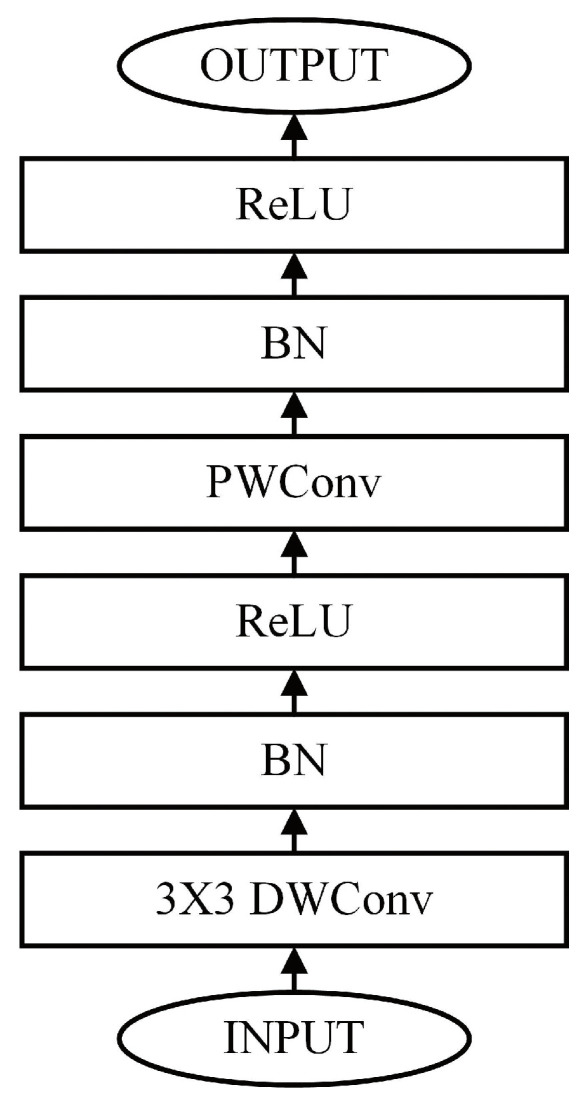
MobileNet Basic Block.

Many CNN models try to merge DSC into their structures, and one of the typical models is MobileNet [24]. The basic component of MobileNet is shown in Figure 2. MobileNet can achieve a top-1 accuracy that is 3% higher on ImageNet compared to AlexNet [25] while its parameters are reduced by 45 times, and the number of operations is reduced by 9.5 times.

**Figure 3:**
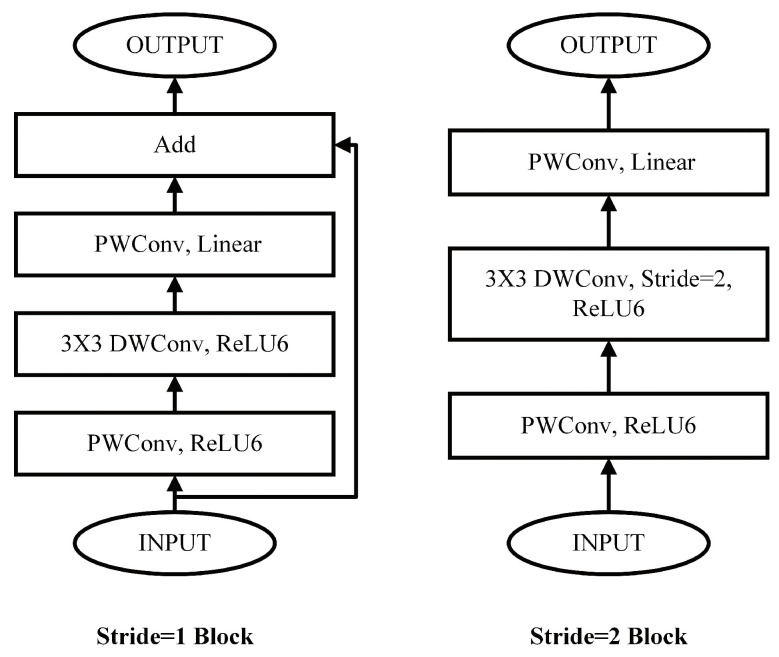
MobileNetV2 basic block.

MobileNetV2 [7], the successor of MobileNet, decreases the number of parameters by reducing some layers’ output channels. It also proposed the Inverted Residuals structure which adds one more PWC before the DSC to improve performance, as shown in Figure 3. In MobileNetV2, the activation function is replaced by ReLU6 to improve robustness. MobileNetV2 can achieve 72% top-1 accuracy on ImageNet with 3.4 M parameters and 300 M MAC operations. Compared to MobileNet, MobileNetV2 achieves a top-1 accuracy that is 1.4% higher and reduces 0.8M parameters. On a Google Pixel 1 phone, the run time of MobileNetV2 is only 75 ms while MobileNet is 113 ms.

## 4. Hardware Design

Since the proposed accelerator aims to fit MobileNetV2’s structures in order to minimize the memory requirement and other resource usages, we build up some specific modules mainly around configurable computation structures and switchable RAM control to achieve this goal. In Section 4.1, the overview of the total design is introduced, and some analysis of different pipeline designs is also given. In Section 4.2, we focus on efficiency, showing the details of the PWC and DWC Accelerators, the choice of the number of input channels for PWC, and some skills to reduce resource usage will be discussed. The remaining sections will focus on memory and dataflow structures. In Section 4.3, two dataflow structures will be discussed and optimized. One is the dataflow between on-chip memory and registers; the other is the dataflow between off-chip memory and on-chip memory. In Section 4.4, a new on-chip memory control method is proposed to minimize the memory capacity. The method of reordering outputs is also discussed.

### 4.1. Architecture Overview

**Figure 4:**
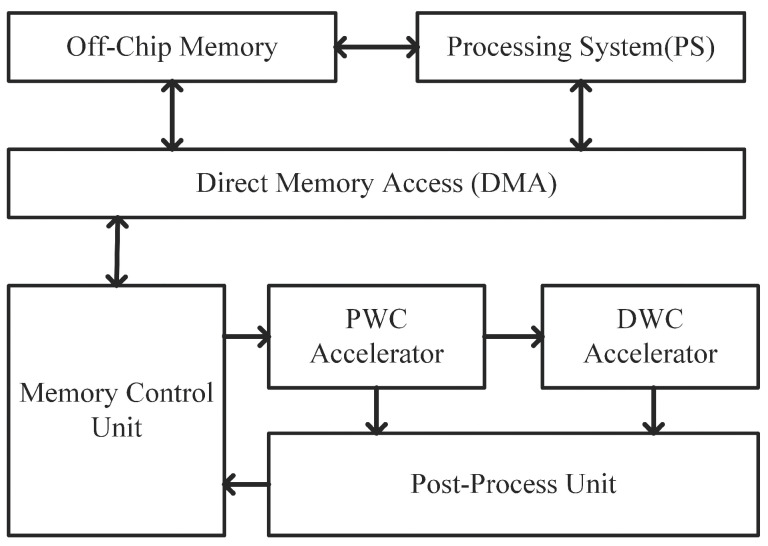
Hardware architecture overview.

The block diagram of the proposed accelerator is shown in Figure 4. It is mainly composed of a Memory Control Unit, the PWC Accelerator, the DWC Accelerator, and a Post-Process Unit. All the parameters and feature maps are stored in off-chip memory initially. The Memory Control Unit controls the data flow, determines which part of data will be stored in specific on-chip memory, and uses a ping-pong buffer while transferring data with off-chip memory to maximize the bandwidth. The PWC Accelerator and the optional DWC Accelerator complete the convolution parallel. Considering the basic structure of MobileNetV2 is a PWC followed by a DWC and then another PWC, adding an additional PWC Accelerator could achieve a full pipeline design. However, an additional PWC Accelerator requests many more resources while only providing a small performance improvement [20]. So, we choose a PWC-DWC pipeline structure to take both performance and resource utilization under consideration. The Post-Process Unit executes other types of operations such as pooling and concatenation. It also generates addresses for results to ensure addresses are continuous.

### 4.2. Compute Engine Design

The main compute engine in the design is the PWC Accelerator and the optional DWC Accelerator because the most common structure in MobileNetV2 is a single PWC and a PWC followed by a DWC.

**Figure 5:**
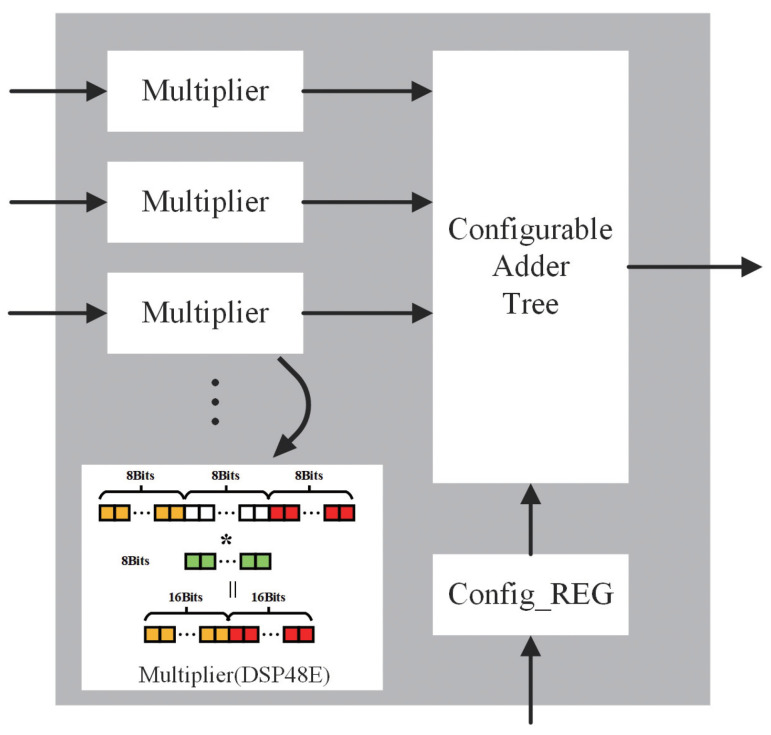
PWC Accelerator architecture.

PWC Accelerator unrolls input channels and output channels to allow parallel calculations, as shown in Figure 5. It can compute 256 multiplications in one cycle at most. Furthermore, the number of channels is changeable in both the implementation stage and runtime stage in order to fit various situations. We achieve this by a parameter in the design stage and a register in the runtime stage. Common designs tend to fix the number of channels to 16 × 16 to keep the balance between input and output bandwidths. However, the fixed 16 input channels cannot be fully utilized at the beginning of a network because the number of channels at the beginning is usually small. Furthermore, the fixed 16 output channels will lead to more resource requirements because we need to increase the scale of all the following parts to meet the requirements of pipeline design. Hence, we design the Configurable Adder Tree to allow the channel number configuration according to the application’s specific situation. Sometimes on-chip memory and the number of logic devices are sufficient. We set a smaller number of input channels and a bigger number of output channels to increase resource utilization, especially during the beginning of a network. Sometimes the memory bandwidth is redundant, but the number of logical devices is not enough, so we can properly increase the number of input channels, such as 32, and the number of output channels reduces to 8. In this way, we reduce the resource usage by 1/2 while doubling the bandwidth usage of on-chip memory. Considering the number of input channels of most CNN layers is bigger than 32, it may lead to lower resource utilization at the beginning of a CNN but the total utilization can be acceptable. Meanwhile, a DSP48E supports a 25 bits multiplier and an 18 bits multiplicand at most. Using one DSP to compute an 8 × 8 multiplication directly will cause more DSP usage. Therefore, we adapt two 8 × 8 multiplications into one DSP48E to reduce the total DSP usage (Figure 5).

**Figure 6:**
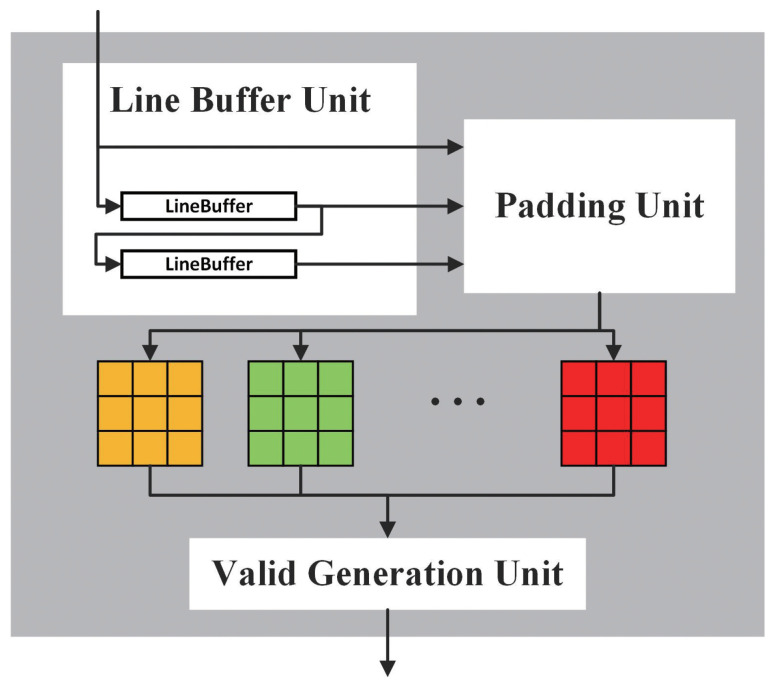
DWC Accelerator architecture.

For the DWC Accelerator shown in Figure 6, many mature and efficient methods for a standard 3×3 convolution have been proposed, such as Winograd [26,27]. Because the DWC part needs to match the PWC output and one kernel is only used in one channel, we keep the data unchanged and feed it into a line buffer unit directly to complete the DWC. The line buffer unit is composed of two connected line buffers. A line buffer could store a whole line of the input feature map and work like a queue, when the first pixel of a new line is pushed, the pixel of the previous line will start to be popped. The line buffer unit allows us to obtain a 3×3 window so that we can operate a 3×3 convolution. Considering that a whole feature map and the weights may be too big to be loaded into buffers, we have to split feature maps and weights into several parts and compute them individually (this is known as loop tiling). So, the DWC Accelerator is designed to add essential extra pixels in the edge of the input feature maps by the padding unit in order to adjust the size of the output feature maps and drop invalid outputs automatically depending on the current input part by the valid generation unit.

### 4.3. Dataflow Structure

In this paper, we optimize the dataflow in order to minimize the data transfer between the on-chip memory and off-chip memory, the on-chip memory and registers, and to minimize the on-chip memory capacity requirement.

First, the on-chip memory and registers are to be discussed. Generally speaking, there are four kinds of dataflow based on their data handling characteristics: Weight Stationary (WS), Output Stationary (OS), Non Local Reuse (NLR), and Row Stationary (RS) [14]. For PWC, which has the largest amount of operations in DSC, using a WS-like dataflow structure could maximize the efficiency according to [28]. For DWC, considering its relatively small amount of operations, we also use a WS-like dataflow structure to simplify the design.

**Figure 7:**
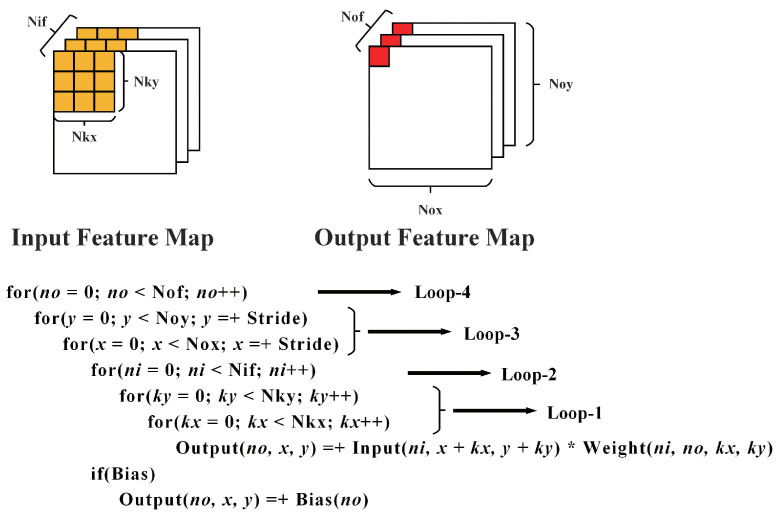
Default standard convolution order.

For on-chip memory and off-chip memory, we want to reduce the access to the off-chip memory since such communication is expensive. Furthermore, the minimum amount of data transfer between the on-chip memory and off-chip memory is reading and writing each data block only once. A standard convolution is performed by four levels of loops shown in Figure 7. The order and unrolling of these loops will influence the data footprint and further determine the data access pattern. We need to find the suitable order and unrolling method, as well as the data tiling approach if the on-chip memory is insufficient.

According to [29], there are three ways to achieve minimal data access: compute Loop-3 first and buffer all required weights for a pixel; compute Loop-4 first and buffer all required pixels for a weight; and buffer all weight or input pixels. The first method is suitable for the situation where input channels can not be fully buffered. However, while tiling, we only split the feature map horizontally because the continuous address is beneficial to DMA transfer, meaning we store all input channels. This method also has a complex control method. The second method conflicts with the WS dataflow. Thus, we minimize data access by storing all weight or input pixels. Specifically, we store the weight in the front half of the network and the input pixels in the second half because the amount of weight will become bigger as the network goes deeper, while the amount of input pixels becomes smaller. For partial sums storage, since we unroll Loop-1, Loop-2, and Loop-4, we set Loop-1 as the innermost loop and then Loop-3 to meet the requirement of the WS dataflow. To minimize the partial sums storage, Loop-1 and Loop-2 need to be performed as early as possible. So, the final order of our convolution is Loop 1-3-2-4.

### 4.4. On-Chip Memory Control Method

Traditional hardware accelerators use two of the same on-chip ping-pong memories as their input and output buffers. This kind of design can maximize the bandwidth. However, in MobileNetV2, using two of the same buffers will waste nearly half of the on-chip memory. This is because MobileNetV2 uses Inverter Residual as the basic block, and the size of PWC input and output is unbalanced. Specifically, Inverter Residual is composed of one expansion PWC, one DWC, and one contract PWC. In DWC, the number of input channels and output channels is equal. However, in the remaining two PWCs, the ratio of output channels and input channels is 6 or 1/6 (6 is the expansion factor in MobileNetV2). Meanwhile, the size of Height and Width of the feature map is not changed. Therefore, the ratio of feature maps’ size is also 6 or 1/6. For two of the same buffers, this unbalanced size means the utilization of one of them will only approach 1/6.

**Figure 8:**
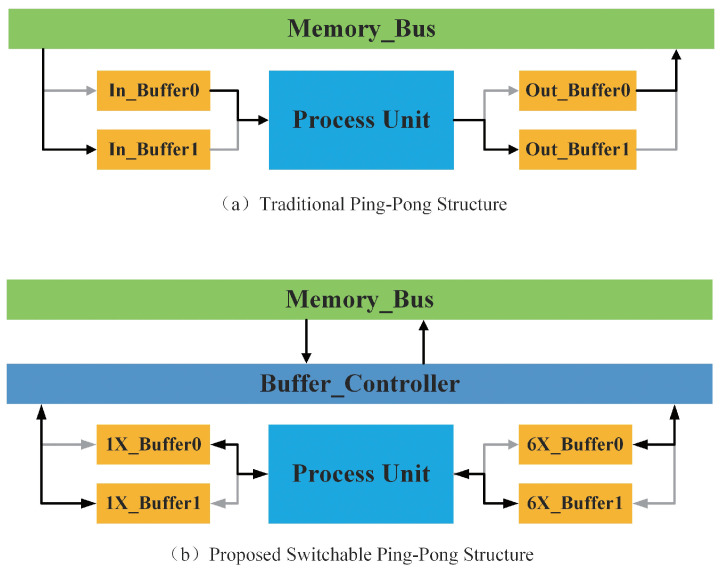
On-chip memory architecture.

In this paper, we proposed a switchable ping-pong RAM to solve this problem, as shown in Figure 8. We set two different size ping-pong buffers, and the ratio of their capacity is 6. The Memory Control Unit will pick one of them as the input buffer and another as the output buffer according to the type of the current layer. In this way, we can save almost half of the memory compared to the two same ping-pong buffers’ structures while doing the same operation under the same tiling.

**Figure 9:**
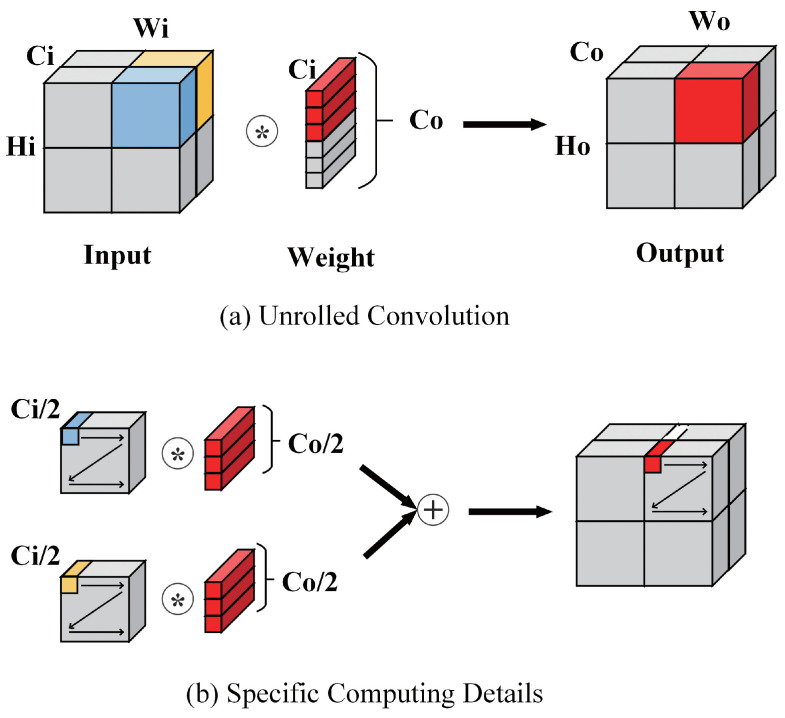
Unrolled convolution and computing details.

Because of the unrolling of output channels, the original outputs are not continuous on physical addresses. As shown in Figure 9a, it assumes that the addresses of the input feature maps are in the order of Channel, Width, and Height; both input channels and output channels are unrolled into two parts. If we want to compute the red part of the output feature map, we need to compute it as shown in Figure 9b. The white block in Figure 9b is the supposed part to be computed next if the output could have the same order as the input. Obviously, the output addresses are not continuous along the Channel. However, we hope the output feature map has the same address order as the input feature map so that we can avoid re-ordering the data after the off-chip memory receives all of the results. Or, we can re-order the data during the DMA transfer, but DMA also requires continuous physical addresses in order to maximize bandwidth. Thus, an address generation module is proposed in the Post-Process Unit in order to generate addresses for the original outputs. This module generates the corresponding final addresses for each output pixel while storing it in the output buffer. In this way, we can ensure the addresses are continuous and have the same order as the input during the DMA transfer and in the off-chip memory.

## 5. Results

The proposed accelerator architecture is demonstrated by implementing the MobileNetV2 network on the Xilinx XC7Z020 FPGA, which contains 13,300 logic blocks, 140 Block RAM, and 220 DSP48E. Each logic block has four 6-input LUTs and eight Flip-Flops. The reason why we choose XC7Z020 and the final implementation results will be described below.

### 5.1. Experimental Framework

**Figure 10:**
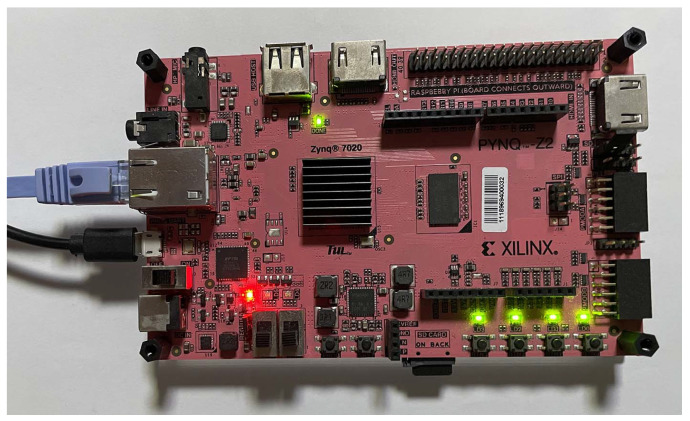
Physical picture of the platform.

Since our goal is minimizing the memory requirement and other resources usages to deploy MobileNets into small devices, we choose Xilinx XC7Z020 FPGA as our target device due to its limited resources. We simulate a real situation. For MobileNetV2, we use the quantization method proposed by [30] to quantize the network to 8 bits. Furthermore, we also fuse each Conv-BN block to reduce the operations and parameters further. The middle results are stored as 32 bits to avoid overflow. For PWC, we use the 32-input and 8-output configuration to minimize resource usage. DWC is configured as 8×8, which matches the PWC. We set the smaller feature buffer to 28,800 bytes, the weight buffer to 18,880 bytes, and the partial sum buffer to 32,768 bytes. DSP is used for multiplications of the PWC part and all quantization processes. Multiplication of the DWC part is executed by LUT due to resource limitations. Since there is a dual-core Cortex-A9 on board, we use it to load all the weights and the input image from the SD card into the DRAM shared by the Cortex-A9 and the FPGA before starting the inference. When data is ready, we send a start signal and the configuration of each layer to finish the inference. The physical picture of our platform is shown in Figure 10.

### 5.2. Experimental Results

The implementation platform is PYNQ-Z2 with Xilinx XC7Z020 FPGA and 512 MB DDR3 as off-chip memory. The standard bandwidth of DDR3 is 1050 Mbps. Dual-core Cortex-A9 is used as the Processing Stream Controller. The bus protocol of IPs is AMBA AXI4 bus. We use the Processing Stream Controller to send only the information of each layer to the accelerator to keep a high efficiency while producing various depthwise separable convolutions. The implementation result is listed in Table 1.

To analyze the performance in detail, we measure the communication and computation delay in each layer. The results are shown in Table 2. The results show that the PWC+DWC parts have shorter communication and longer computation delays compared with the PWC in the same layer. This makes sense because the PWC+DWC parts perform the channel expansion, meaning that the amount of input data is small. So, the computation could start quickly when one of ping-pong RAMs is loaded. We can see the communication delay of the PWC in the 4th, 5th, and 6th lines are bigger than others. This is because we store all the weights in order to minimize the off-chip access in these layers when the sizes of the input feature map are small enough to be buffered. So, we also store all the input feature maps without tiling to simplify the computation in these layers, which may cause longer communication times.

The reduction in the resource requirement of the proposed method is significant when using the 32 × 8 PWC module mentioned in Section 4.2 and the memory control method mentioned in Section 4.4. Otherwise, it is impossible to deploy the accelerator on the target platform under the same situation due to P&R congestion and over-required memory and logic resources. Table 3 provides a comparison between the solution proposed in this work and other similar ones. The final result shows that we use the lowest memory resources while achieving a high efficiency compared with other implementations. Due to resource limitations, we cannot achieve a very high speed, but we achieve the highest efficiency according to FPS/MHz per DSP per KB. In the future, we will try to increase the frequency and to further reduce the storage of the partial sum in order to achieve a higher performance.

## 6. Conclusions

In this work, a high-performance, low-memory utilization CNN accelerator is proposed. This structure is optimized for depth separable convolution, especially MobileNetV2. As the implementation sample described above, we deploy our accelerator on Xilinx XC7Z020 and achieve 70.94 FPS under 150 MHz while only using 524.25 KB on-chip memory and 176 DSPs. This means that our design is sufficient for auxiliary medical applications in both speed and resource requirements. Compared to other similar designs, we use the least on-chip memory and achieve a relatively high efficiency according to the FPS/MHz per DSP. Due to the two-layer pipeline structure that is mentioned in Section 4.1, our design cannot achieve the highest efficiency. However, it helps us to reduce a large amount of on-chip memory usage and still keeps a relatively high efficiency. Therefore, the proposed structure matches our goal and can be fit into auxiliary medical devices with high performance and can run in real-time. 

## Figures and Tables

**Table 1:** Resource utilization.

Name	LUT	DSP (DSP48E)	BRAM
Accelerator	32,956 (61.9%)	176 (80%)	107.5 (76.8%)
DMA	2000 (3.8%)	0 (0%)	12 (8.6%)
Other	1333 (2.5%)	0 (0%)	0 (0%)
Total	36,289 (68.2%)	176 (80%)	119.5 (85.4%)

**Table 2:** Communication and computation delays in each layer.

Layer (Input Size)	Operator	Stride	PWC+DWC		PWC ${}^{1}$	
			Communication Delay (us)	Computation Delay (us)	Communication Delay (us)	Computation Delay (us)
224 × 224 × 3	Conv2d	2	N/A	N/A	24.56	405.81
112 × 112 × 32	Bottleneck	1	31.40	586.67	24.56	404.49
112 × 112 × 16	Bottleneck	2	34.01	1296.53	65.02	270.47
		1	30.65	600.87	108.00	430.33
56 × 56 × 24	Bottleneck	2	30.65	522.08	106.08	129.47
		1	24.07	265.79	127.01	150.92
28 × 28 × 32	Bottleneck	2	24.07	171.47	33.04	78.68
		1	16.55	209.50	65.68	144.35
14 × 14 × 64	Bottleneck	1	16.55	209.47	65.79	216.36
		1	24.71	412.38	98.43	314.83
14 × 14 × 96	Bottleneck	2	24.71	341.67	28.08	152.12
		1	21.43	309.20	46.32	244.99
7 × 7 × 160	Bottleneck	1	21.43	309.17	46.85	489.72
7 × 7 × 320	Conv2d	1	N/A	N/A	19.65	670.02
1280 × 1000	Conv2d	1	N/A	N/A	9.77	1345.96

^1^ Include pooling or concatenation if necessary.

**Table 3:** Compared with other implementations.

	[19]	[20]	[21]	[31]	This Paper
Platform	Arria10	ZU2	Arria10	Virtex7	XC7Z020
CNN Model	MobileNetV2				
Frequency	133 MHz	430 MHz	200 MHz	150 MHz	150 MHz
DSP Usage	1278	212	1220	2160	176 (248 ^1^)
On-Chip Memory Usage	1844 M20K (3.07 MB)	145 BRAM	15.3 Mb (1.91 MB)	941.5 BRAM	**119.5** BRAM (**524.25** KB)
Speed	266.2 FPS	205.3 FPS	**1050** FPS (Throughput)	302.3 FPS	70.94 FPS
FPS/MHz per DSP per KB ($10^{-6}$)	0.50	3.54	2.20	0.23	**5.13 (3.64 ^1^)**

^1^ As mentioned above, we use LUTs to conduct the multiplications in DWC because of insufficient resources. This will cause unfair results. Here, we assume every multiplication is conducted by DSPs and calculate the relevant results.

## Data Availability

Data sharing not applicable.
